# Detection of interacting transcription factors in human tissues using predicted DNA binding affinity

**DOI:** 10.1186/1471-2164-13-S1-S2

**Published:** 2012-01-17

**Authors:** Alena Myšičková, Martin Vingron

**Affiliations:** 1 Max Planck Institute for Molecular Genetics, Ihnestr. 73, 14195 Berlin, Germany

## Abstract

**Background:**

Tissue-specific gene expression is generally regulated by combinatorial interactions among transcription factors (TFs) which bind to the DNA. Despite this known fact, previous discoveries of the mechanism that controls gene expression usually consider only a single TF.

**Results:**

We provide a prediction of interacting TFs in 22 human tissues based on their DNA-binding affinity in promoter regions. We analyze all possible pairs of 130 vertebrate TFs from the JASPAR database. First, all human promoter regions are scanned for single TF-DNA binding affinities with TRAP and for each TF a ranked list of all promoters ordered by the binding affinity is created. We then study the similarity of the ranked lists and detect candidates for TF-TF interaction by applying a partial independence test for multiway contingency tables. Our candidates are validated by both known protein-protein interactions (PPIs) and known gene regulation mechanisms in the selected tissue. We find that the known PPIs are significantly enriched in the groups of our predicted TF-TF interactions (2 and 7 times more common than expected by chance). In addition, the predicted interacting TFs for studied tissues (liver, muscle, hematopoietic stem cell) are supported in literature to be active regulators or to be expressed in the corresponding tissue.

**Conclusions:**

The findings from this study indicate that tissue-specific gene expression is regulated by one or two central regulators and a large number of TFs interacting with these central hubs. Our results are in agreement with recent experimental studies.

## Background

Transcriptional regulatory networks determine a spatiotemporal variance in gene expression which enables the tissue-specificity of the cell [1]. Regulatory networks include groups of control proteins, such as transcription factors (TFs) binding to short DNA motifs, called transcription factor binding sites (TFBS). Each TF can be connected to a set of its target genes - genes on whose promoters the TF binds in order to activate or repress them [2]. In mammalian tissues, TFs do not usually act alone but form complexes with other TFs and co-factor proteins, which bind together to the DNA synergistically to affect the transcription of the target genes [3]. This combinatorial regulation increases the specificity and flexibility of genes in controlling tissue development and differentiation. Therefore, detection of interacting TFs can significantly increase our understanding of how tissue specificity is determined.

Over the last years, a variety of experimental approaches was introduced to detect TF interactions controlling tissue gene expression. Among the most used technologies, gel retardation assays [4], genomic microarrays [5], or chromatin immunoprecipitation followed by microarrays or high-throughput sequencing [6,7] were used to construct transcriptional models in different tissues. However, these studies are able to detect TF interactions on a limited scale since they treat each TF separately. A novel two-hybrid screening method which can detect physical protein-protein interactions was applied in mouse and human [8,9]. Nevertheless, such technology is able to detect just a part (25%) of all possible TF interactions [9].

To overcome the experimental limitation, several computational models were built to predict tissue-specific interacting TFs. Some of these models combine gene expression information with promoter sequence features [10-12] or integrate the evolutionary conservation of TFBS on promoters of tissue-specific genes [13]. However, the results of these studies can be biased by pairs of cooperating TFs with similar motifs, as discussed in [14]. Comparing all these methods shows that just a small fraction of predicted TFs interactions can be found in more than one study. This suggests that different methods are able to identify interacting TFs from different perspectives and that the mechanism regulating the tissue differentiation and development is still not completely understood. With our study we aim to create the next component in understanding the transcriptional networks in human tissues. To identify interacting TFs, we combine the predicted binding affinities of TFs on their target genes while investigating all possible pairs of studied TFs with the hypergeometric test. Furthermore, we include information about the tissue-specificity of the target genes and apply a 3-way contingency table test to determine the significance of the overlap of tissue-specific top-ranked target genes for pairs of different TFs. Our approach is based on the following two assumptions. First, two interacting TFs are expected to share a significant number of their target genes in comparison with two randomly selected TFs. Second, the list of target genes of a single TF can be represented by a ranked gene list based on the binding affinity of the TF to the promoter sequences. To our knowledge, this is the first method which is able to predict interacting TFs based only on predicted TF-binding affinity to the promoter sequence and its tissue-specificity information.

## Methods

### Similarity of ranked lists of target genes measured by the hypergeometric test

In our model, we use a simple assumption that two interacting TFs should share a significant number of identical target genes. In other words, if two different TFs bind on the same promoter regions they would very likely act together to direct the expression of their target genes. To evaluate the significance of the shared target genes, we apply the hypergeometric test for ranked lists of a TF's target genes.

First we define the human promoter regions as -500 - 0 bp relative to the transcription start site (TSS) from Ensembl GRCh37/hg19 assembly of the human genome [15], [http://genome.ucsc.edu]. To create the ranked list of target genes we first scan all such human promoter regions with TRAP predictor [16]. We choose the TRAP approach since it avoids the artificial separation between binding sites and non-binding sites but instead calculates the binding probability of a given TF to all sites in the sequence based on a biophysical model.

The binding affinity of all 130 TFs, represented by position weight matrices (PWMs), in the JASPAR CORE Vertebrata database [17] to all human promoters is calculated. Separately for each TF, we rank the promoter regions by their binding affinity in a decreasing order, such that the genes with high binding affinity are placed at the top of the list. We measure the similarity of these ranked lists for all possible pairs (130 * 129/2 = 8385) of TFs by calculation of the significance for the shared target genes among the top-*L*_1 _(for the first TF) and the top-*L*_2 _(for the second TF) ranked genes using the hypergeometric test [18]. This problem corresponds to a simple 2-way contingency table with two indicator random variables *X *and *Y*. Variable *X *indicates genes ranked among the top-*L*_1 _in the list of the first TF and variable *Y *indicates genes ranked among the top-*L*_2 _in the target gene list of the second TF. The hypergeometric test was used in a previous study [19] to predict protein-protein interactions (PPIs) in yeast based on shared protein neighbors in small world interactions.

To estimate the best performing thresholds *L*_1 _and *L*_2 _we repeat the testing procedure for varying values of both cutoff points: *L*_1_, *L*_2 _∈ {10, 20, ..., 990, 1000} which correspond to 10^4 ^possible combinations. We assume that the smallest obtained *p*-value of the hypergeometric test is associated with the highest similarity between the two rank lists of target genes. A similar technique was applied by Roider *et al. *[20] to identify significant association of tissue specific genes and target genes of transcription factors.

### Confounding factor: motif similarity

When two TFs have very similar motifs (represented by PWMs), with high probability their ranked lists of target genes will be very similar [14]. To eliminate the choice of candidates which would share a significant number of the identical genes in the top of the lists due to their similar matrices (and not necessarily due to their real co-occurrence), we include a confounding factor into the analysis, a motif similarity measure. For all pairs of TFs, we calculate their motif similarity using the MOSTA *S*^max ^similarity measure [21], which is based on the log-odds ratio of the overlap probability and the independent probability of hits of the two motifs on both strands of a DNA sequence.

The similarity measure for all TF pairs ranges from -1.12 to 8.58. To avoid the presence of TF interactions with highly similar motifs in our predictions, we concentrate on TF pairs with motif similarity smaller than four. This cutoff corresponds to the 90%-quantile of the empirical distribution of *S*^max ^and avoids the choice of significantly similar motifs in the JASPAR database.

### Similarity of ranked lists of target genes in a tissue measured by testing in 3-way contingency tables

By definition, a 2-way contingency table depicts the association of two variables. In our case, the two variables come from two TFs. In order to stratify by tissue, we need to introduce a third dimension, thus arriving at a 3-way contingency table. We introduce variable *Z*_*t*_, an indicator function for genes specific in the tissue *t*:

$$Z_{t}\left(i\right)=\left\{\begin{array}{cc} 1 & \text{gene}\;i\;\text{specific}\;\text{for}\;\text{tissue}\;t\\ 0 & \text{otherwise}. \end{array}\right)$$

**Figure 1:**
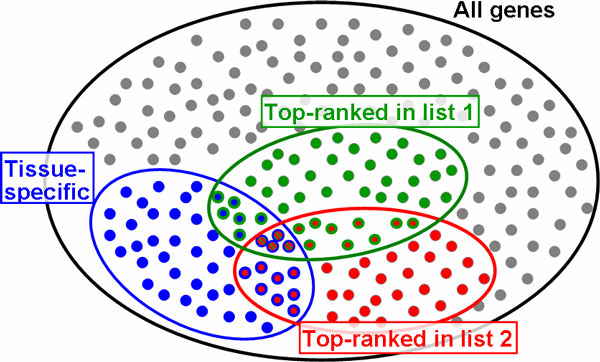
**Venn diagram.** Venn diagram of the setting for independence tests in 3-way contingency tables. Grey dots indicate all human genes, blue dots are genes known to be specific for a selected tissue. Green and red sets denote the top-ranked target genes of the first and second TF, respectively.

As in the previous section, random variables *X *and *Y *indicate genes ranked among the top-*L*_1 _and top-*L*_2 _in the list of the first and second TFs, respectively. A graphic illustration of this situation is shown in Figure 1. All human genes are shown as dots, blue ones indicate tissue specific genes (where *Z*(*i*) = 1). The green set highlights the top-ranked target genes of the first TF (*X*(*i*) = 1) and the red set highlights the top-ranked target genes of the second TF (*Y*(*i*) = 1). The corresponding 2 × 2 × 2 contingency table is shown in Table 1.

**Table 1:** 2 × 2 × 2 contingency table

**Genes with **...	Tissue specificity		No tissue specificity		Sum
	**Rank ≤ *L***_**2**_	**Rank >*L***_**2**_	**Rank ≤ *L***_**2**_	**Rank >*L***_**2**_	
Rank ≤ *L*_1_	*μ*_111_	*μ*_121_	*μ*_112_	*μ*_122_	*μ*_1++_
Rank >*L*_1_	*μ*_211_	*μ*_221_	*μ*_212_	*μ*_222_	*μ*_2++_

Sum	*μ*_+11_	*μ*_+21_	*μ*_+12_	*μ*_+22_	*μ*_+++_

2 × 2 × 2 contingency table for shared genes among the top-*L*_1 _and top-*L*_2 _ranked target genes of two different TFs and tissue-specific genes.

To test whether the number of genes in the intersection of all 3 variables, e.g. *μ*_111 _:= ∑_*i*_(*X*(*i*) = 1, *Y*(*i*) = 1, *Z*(*i*) = 1), is larger than expected by chance, a 3-way contingency table test is applied [22]. There are 3 possible hypotheses to be formulated in a 3-way contingency tables: (a) mutual independence of *X*, *Y *and *Z*, (b) conditional independence of *X *and *Y *given *Z *and (c) partial independence of composite *XY *and *Z*. The expected frequencies in the contingency table are estimated depending on the tested hypothesis. In our case we would like to detect such TF pairs, which *share *a significant number of target genes in a tissue. This corresponds to the partial independence hypothesis (c).

The expected frequencies under the null hypothesis in the 2 × 2 × 2 contingency table are defined as follows:

$${\hat{\mu }}_{xyz}=\frac{\mu_{++z}\mu_{xy+}}{n};\;x,y,z\in \left\{0,1\right\}.$$

Here, *μ*_++__*z *_denotes the one-way marginal of *Z *defined as $\mu_{++z}=\underset{x,y=\left\{0,1\right\}}{\sum }\mu_{xyz}$ for *z *∈ {0,1}. *μ*_*xy*__+ _denotes the *xy*-two-way marginal, and in the same way: $\mu_{xy+}=\underset{z=\left\{0,1\right\}}{\sum }\mu_{xyz}$ for *x*, *y *∈ {0, 1}.

The test statistic for 3-way contingency tables is defined as the log-likelihood ratio of observed (*μ*_*xyz*_) and expected frequencies (${\hat{\mu }}_{xyz}$) over the groups of variables *X*, *Y *and *Z *[22]:

2I(μ:μ^)=2∑x,y,z={0,1}μxyz logμxyzμ^xyz~χdf2.

*df *denotes the degrees of freedom of the *χ*^2 ^distribution and equals 3 for this particular test (one degree of freedom for each variable for which expected frequencies have to be estimated). The test statistic can be calculated simply using the loglinear representation [22].

## Results

### Detected interactions by the hypergeometric test

**Figure 2:**
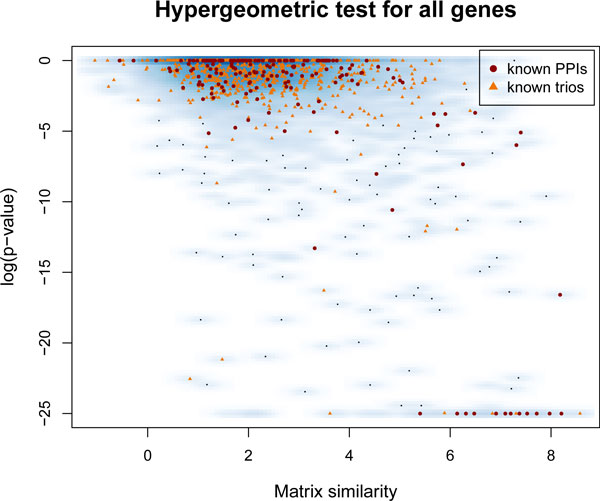
**Smooth scatterplot of motif similarity measure and *p*-values of the hypergeometric test.** Logarithm of the smallest *p*-values of the hypergeometric test for all tested TF pairs (vertical axis) vs. motif similarity measure *S*_max _(horizontal axis). Red points and orange triangles denote experimentally shown PPIs and trios with a known interacting co-factor, respectively.

To assess the association between the similarity of ranked lists and the similarity of PWMs, we study the relation between the smallest *p*-values obtained from the hypergeometric test and the PWM similarity measure *S*^max ^(smoothed density scatterplot in Figure 2). As expected, TF pairs with very similar motifs (*S*^max ^∈ [6,8]) correspond to highly significant *p*-values (data cloud in lower right corner). We identify already known PPIs from the FANTOM Consortium [9] and BioGRID database [23]; and those TF pairs which have the same known co-factor (trios) found in these databases [9,23] (Figure 2, red dots and orange triangles, respectively). However, the majority of these known interactions correspond to TF pairs with rather low significance (log_10 _*p *∈ [-3; 0]).

**Figure 3:**
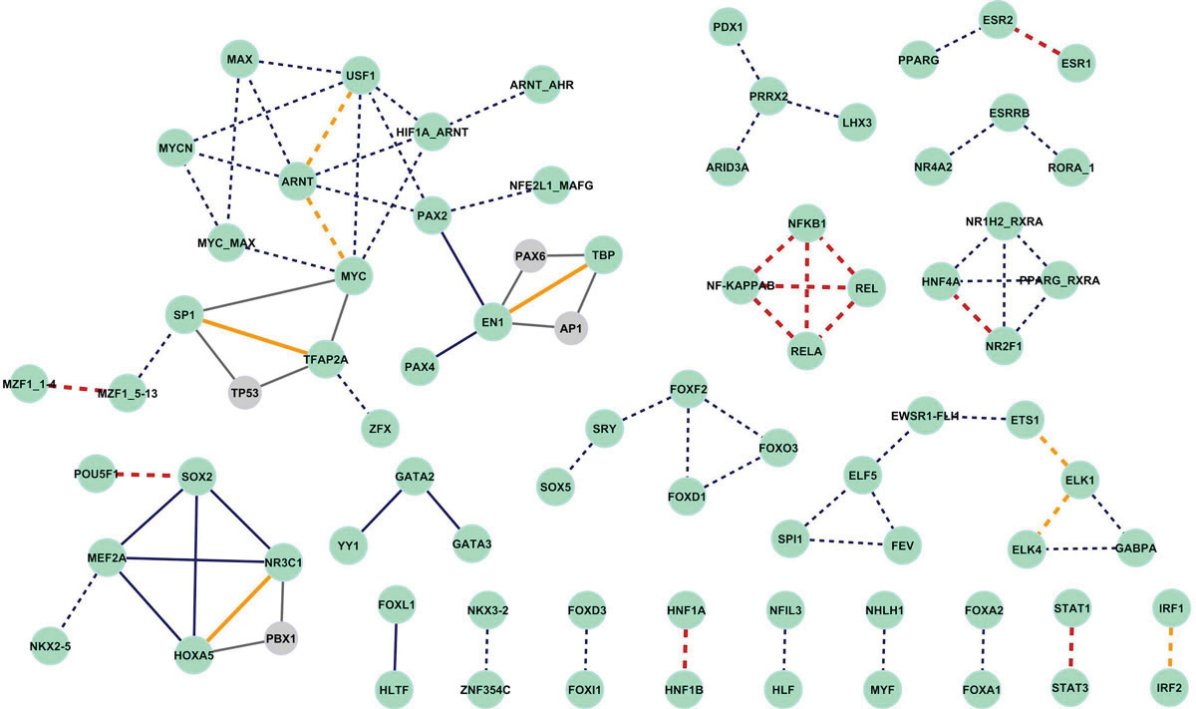
**Predicted network of TF interactions.** Network of the predicted TF interactions based on the 2-dimensional hypergeometric test. Red and orange edges indicate known PPIs and known trio interactions, respectively. Solid lines denote interactions between TFs where *S*^max ^< 4, dashed lines indicate interactions between TFs with *S*^max ^≥ 4. Common co-factors which were included in the network but were not predicted are denoted by grey color.

In Figure 3, TF pairs with *p*-value ≤ 10^-20 ^are shown. The network consists of 76 interactions, of which 15.8% were found to be known PPIs (denoted as red edges). 22.4% are known trios, highlighted in orange. Among those, we focus on 13 interactions between TF pairs with low motif similarity (*S*^max ^< 4) which are represented by solid lines. Three TF pairs have one or more common co-factor (EN1:TBP interacts with AP1 and PAX6; SP1:TFAP2A with TP53 and HOXA5:NR3C1 with PBX) which are indicated as grey nodes with corresponding grey edges. The evidence of a common third co-factor increases the probability that these TFs can interact on the promoter. Manke *et al. *[24] showed that the TFs build networks mostly with a length of 2-4 molecules. Further, we find with IPA software developed by Ingenuity (Redwood City, CA, USA) an experimental confirmation of our predictions in the literature for these two interactions: SP1:TFAP2A [25-27] and GATA2:GATA3 [28].

### Prediction of tissue-specific interactions

Before applying the new statistical test, tissue-specific genes have to be defined. For our analysis we use the data from Yu *et al. *[29] for 30 human tissues and data from Haas *et al. *[30] for 4 homogenous tissues. Both are based on expression enrichment values of EST clusters in tissues. We prefer data based on the ESTs analysis rather than microarray expression data which show much higher variability in their measurements. The number of tissue-specific genes varies from 58 (uterus) to 1409 (lymphocyte) which are small numbers in comparison with the total number of promoters (42380 in GRCh37/hg19 assembly of the human genome [15]).

To avoid multiple testing problems we fix the length of top-ranked target genes to 1000 for all TFs and do not repeat the testing procedures with various thresholds as in the 2-way contingency tables. The cutoff of 1000 genes is justified by small numbers of tissue-specific genes and large number of promoters. Applying different values of this threshold changes the scale of the *p*-values but not their ranking. Using the first 1000 top-ranked target genes, the expected number of shared top-ranked tissue-specific genes for two different TFs can vary between 0 and 35.

In total, we identify 594 significant TF pairs in 4 specific cell lines (*p*-value ≤ 10^-11^) and 409 significant TF pairs in 12 human tissues (*p*-value ≤ 10^-6^). 869 (86.6%) of these interactions are between TFs with nonsimilar motifs (*S*^max ^< 4). The most interactions are found in retinal pigmented epithelium (259), the least (1) in stomach. 181 TF pairs are significant in two or more different tissues, 61 of them are common for kidney and liver and 43 are common for hematopoietic stem cells and lymphocytes. There are no significant interactions with the threshold of *p*-value ≤ 10^-6 ^in 18 tissues. We find additional 58 interactions with larger *p*-value ∈ (10^-6^, 10^-5^], 17 of them in another 6 tissues. The tissue-specific interactions in all of the 22 tissues are summarized in Table 2. All predicted interactions including motif similarity measure and *p*-values are listed in Additional file 1.

**Table 2:** Summary of predicted tissue-specific TF pairs with 3 most significant TF pairs in 22 human tissues

Tissue	# interactions (nonsimilar)	# factors	Top three nonsimilar interactions	Hubs
Bladder*	3 (3)	3	ELK1:NFYA, ELK1:NOBOX, NFYA:NOBOX	-
Blood	6 (4)	5	SPI1:ARID3A, SPIB:ARID3A, SPI1:CTCF	ARID3A, SPI1, SPIB
Bone	24 (24)	25	TBP:TFAP2A, TBP:EWSR1-FLI1, TBP:NOBOX	TBP
Brain	25 (17)	20	SP1:SOX10, SP1:ESR2, SP1:REST	MZF1, SP1
Cervix	40 (30)	24	ZFP423:ZFX, ELK1:ZFX, MIZF:ZFX	ZFX, KLF4, ZFP423
Eye*	4 (2)	6	T:HNF1B, SP1:TAL1-TCF3	SP1
Heart*	6 (5)	7	MEF2A:MAFB, MEF2A:NFKB1, MEF2A:REST	MEF2A
Kidney	95 (87)	64	GATA1:HNF1A, HNF1A:ARID3A, TP53:HNF1B	HNF1A, HNF1B
Liver	106 (99)	67	HNF1A:HNF1B, HNF1A:HNF4A, HNF1A:CEBPA	HNF1A, HNF1B
Lymph node	64 (57)	65	SPI1:MZF1, SPI1:MYF, SPI1:FOXQ1	SPI1
Muscle	41 (38)	40	MEF2A:ZFP423, MEF2A:NHLH1, MEF2A:NFIC	MEF2A, TBP
Pancreas*	14 (13)	15	AR:TAL1-GATA1, MZF1:TAL1-GATA1, E2F1:TAL1-GATA1	TAL1-GATA1
Placenta*	2 (2)	4	RREB1:PDX1, ESRRB:POU5F1	-
PNS*	1 (0)	2	ELK4:REL	-
Sm. intestine*	1 (1)	2	NFYA:TBP	-
Stomach*	7 (6)	8	EWSR1-FLI1:PLAG1, MYC:PLAG1, PAX6:PLAG1	PLAG1
Testis*	16 (14)	19	FOXC1:HOXA5, ARNT-AHR:NOBOX, ARNT-AHR:NKX2-5	ARNT-AHR
Tongue*	12 (11)	16	NFKB1:NFIL3, NFKB1:TFAP2A, GATA3:NKX3-1	NFKB1
Adipose**	104 (90)	46	MZF1:NFYA, NFYA:MYB, NFYA:TBP	NFYA, MZF1
Lymphocyte**	181 (156)	107	ELK1:CEBPA, ELK1:FOXA2, ELK1:POU5F1	NFYA, ELK1, GABPA
Hematop. SC**	50 (41)	36	ELK1:NFYA, NFYA:GABPA, ELK1:EGR1	NFYA, ELK1
Retinal pigm. epithelium**	259 (219)	116	ARNT-AHR:CREB1, ARNT-AHR:NFYA, CREB1:BRCA1	CREB1, NFYA, PAX2

* Network predicted with *p *≤ 10^-5^, ** network predicted with *p *< 10^-10^.

Summary of predicted tissue-specific TF pairs (with *p*-value ≤ 10^-6^) in 22 human tissues.

### Evaluation by known protein-protein interactions

**Figure 4:**
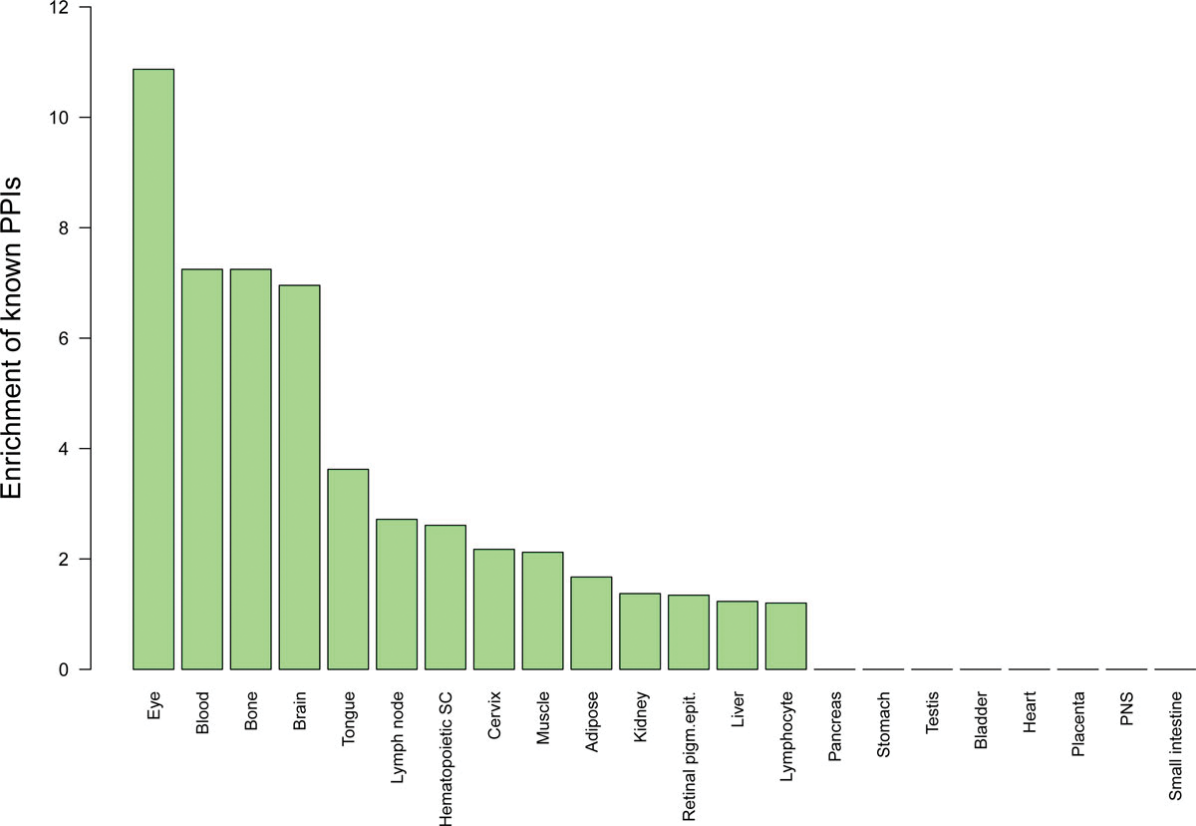
**Enrichment of known protein-protein interactions.** Enrichment of known protein-protein interactions among predicted TF interactions in 22 tissues.

To evaluate our predictions, we calculate the ratio of experimentally validated PPIs from FANTOM and BioGRID databases [9,23] in the set of our candidates. 15.8% (6.8-fold enrichment, Fisher's exact test: *p *= 1.6 · 10^-7^) of interactions predicted with the iterative hypergeometric test were found in the protein database. 4.2% (1.8-fold enrichment, Fisher's exact test: *p *= 8.4 × 10^-4^) of predicted tissue-specific interactions are already validated PPIs. Further we calculate the enrichment of known PPIs among the candidates for each tissue, shown in the bar plot in Figure 4. Whereas for some tissues the percentage of known PPIs is 10- or 7-fold higher than expected by chance (eye, blood, bone and brain), there are 8 tissues (bladder, pancreas, stomach, testis, heart, placenta, peripheral nervous system and small intestine) where no database PPIs were found. The reason for this may lie in the incompleteness of the experimental databases. Usually, there are groups of well-studied proteins and TFs for which many interactions are experimentally validated. Moreover, there are many TFs for which the yeast-2-hybrid experiment cannot be performed due to technical difficulties.

### Predicted interactions in liver

In the next sections we present and validate our predictions of TF interactions in liver, skeletal muscle and hematopoietic stem cells - three well-studied homogenous human tissues for which sufficient information is provided in the literature.

**Figure 5:**
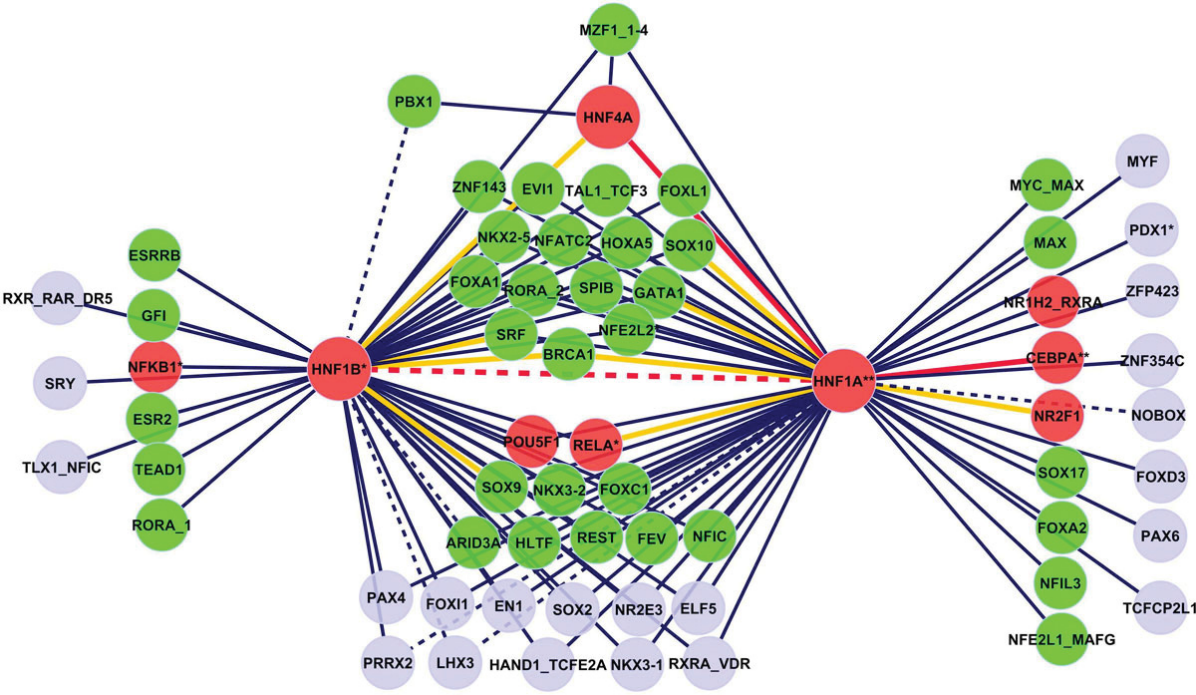
**Predicted network of interactions in liver.** Network of predicted TF interactions in liver based on testing in 3-way contingency tables. Red nodes denote previously known regulators in liver, green nodes indicate TFs expressed in liver. TFs with known function in liver are labeled with an asterisk. Red and orange edges denote known PPIs and known trios, respectively.

The relationship between the *p*-values of the 3-way contingency table test and PWM similarity measure changes due to the stratification by tissue (see Additional file 2). Now, there is a group of highly significant TF pairs with nonsimilar binding motifs. Taking the TF pairs with threshold *p *≤ 10^-6^, 106 interactions among 67 TFs are detected in liver (network shown in Figure 5). Solid edges indicate 98 interactions between TFs with low motif similarity, remaining edges are between TF pairs with high motif similarity. Nine (13.4%) TFs in the network (CEBPA, HNF1A, HNF1B, HNF4A, NR2F1, NFKB1, POU5F1, RELA, RXRA) are supported in the literature to be transcriptional regulators in liver (TRANSFAC database [31,32], IPA Ingenuity Systems). We find 3 (HNF1, HNF4 and CEBP) out of 4 critical regulators from Krivan and Wasserman [33] in our liver regulatory network. The central regulators (HNF1A, HNF4A) from Odom *et al. *[6] are the central hubs with the highest number of interactions in our predicted network. Moreover HNF1A and HNF4A were identified as specifier (high specificity expression) hubs by the experimental work of FANTOM Consortium [9]. The majority (59.7%) of nodes (green color in Figure 5) have experimental evidence supporting expression in liver tissue [9,32,34]. We detect 3 already known interactions between the liver regulators (HNF1A:HNF1B, HNF1A:HNF4A, HNF1A:CEBPA), highlighted with red edges. 9 predicted interacting TF pairs share a common co-factor (orange edges). HNF1A and SOX10 both interact with CEBPA; HNF1A and NR2F1 both interact with HNF4A, which support the hypothesis that these TF pairs will interact too. Next, we search with IPA Ingenuity Systems for enriched functions of the predicted TFs in liver. Among transcriptional regulation and DNA-binding, development of liver (*p *= 1.37 × 10^-06^, CEBPA, HNF1A, HNF1B, PDX1, RELA), proliferation of hepatocytes (*p *= 5.71 × 10^-04^, CEBPA, HNF1A, NFE2L2, NFKB1) and liver hepatitis (*p *= 1.31 × 10^-02^, ESR2, NFE2L2, PDX1, RELA) were found. Factor NFE2L2 is a regulator in lipid metabolism and hepatic system development (4.77 × 10^03^; 9.52 × 10^-03^); RELA factor regulates the degeneration of liver (4.22 × 10^-02^) and we predict that both factors interact with two central liver regulators HNF1A and HNF1B. Known regulatory functions in liver of NFE2L and RELA indicate possible interactions with the central regulators.

### Predicted interactions in skeletal muscle

**Figure 6:**
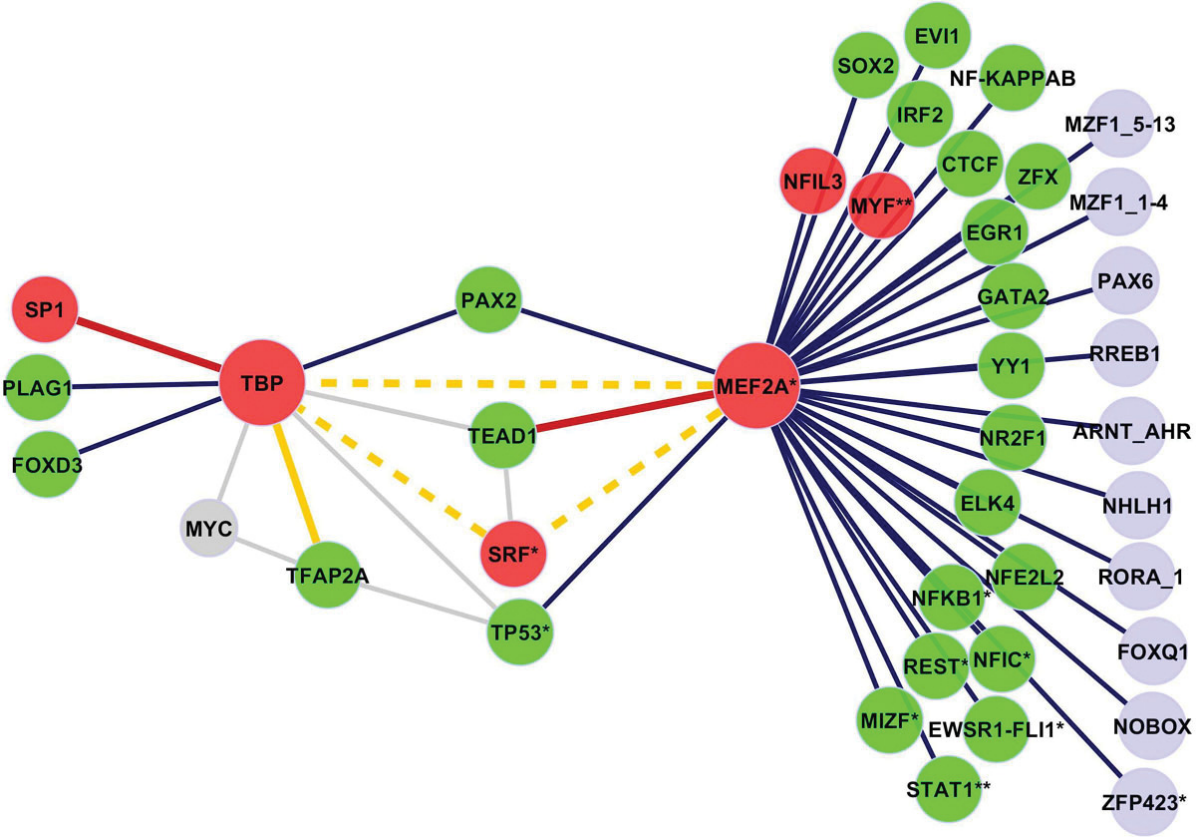
**Predicted network of interactions in skeletal muscle.** Network of predicted TF interactions in skeletal muscle based on testing in 3-way contingency tables. Red nodes denote previously known regulators in skeletal muscle, green nodes indicate TFs expressed in skeletal muscle. TFs with known function in muscle are labeled with an asterisk. Red and orange edges denote known PPIs and known trios, respectively. Common co-factors which were included in the network but were not predicted are denoted by grey color and the corresponding interactions by grey edges.

Figure 6 shows the network with 41 predicted interactions among 40 TFs in skeletal muscle. Here, six TFs (MEF2A, MYF, NFIL3, SP1, SRF, TBP) are known to regulate the gene expression in muscle [11]. MEF2A is the central regulator with the highest number of predicted interactions in our network, TBP is a center of a smaller network related to general tissue development. Both of them were classified as facilitator (widespread expression) hubs by FANTOM Consortium [9]. For 67.5% of factors evidence of expression in muscle is found [9,32,34]. 2 already known PPIs (MEF2A:TEAD1;TBP:SP1) are detected in our predicted network in muscle. Four of the predicted interactions are identified as known trios, which increases confidence in the validity of our predictions. TBP and TFAP2A have two known co-factors: MYC and TP53 (shown as grey edges). Predicted TF pairs SRF:TBP, SRF:MEF2A and TBP:MEF2A all interact with factor TEAD1 (grey edges). Since SRF, TBP and MEF2A are regulators in muscle, there is a high probability that TEAD1 can have a regulatory function in muscle, too. Furthermore we found experimental evidence of physical interaction between SRF and MEF2A in mouse [35]. 8 TFs in the network control the differentiation of muscle cells (*p *= 9.4 × 10^-09^; MIZF, MEF2A, MYF5, NFIC, REST, SRF, STAT1, TP53); 6 TFs in the network are involved in differentiation of muscle cell lines (*p *= 8.1 × 10^-08^; EWSR1, FLI1, MYF5, NFKB1, STAT1, ZNF423). Functional analysis was performed with IPA Ingenuity Systems. Two other functional categories, apoptosis of fibroblast cell lines (*p *= 1.31 × 10^-09^; AHR, EGR1, EVI1, EWSR1, FLI1, NFE2L2, NFKB1, RELA, STAT1, TP53) and development of organs (*p *= 8.5 × 10^-20^; AHR, ARNT, EGR1, EVI1, FLI1, FOXD3, FOXQ1, GATA2, NFKB1, NOBOX, NR2F1, PAX2, PAX6, PLAG1, RELA, RORA, SOX2, SP1, SRF, TEAD1, TFAP2A, TP53, YY1, ZFX, ZNF423), are related processes which occur in muscle.

### Predicted interactions in hematopoietic stem cells

**Figure 7:**
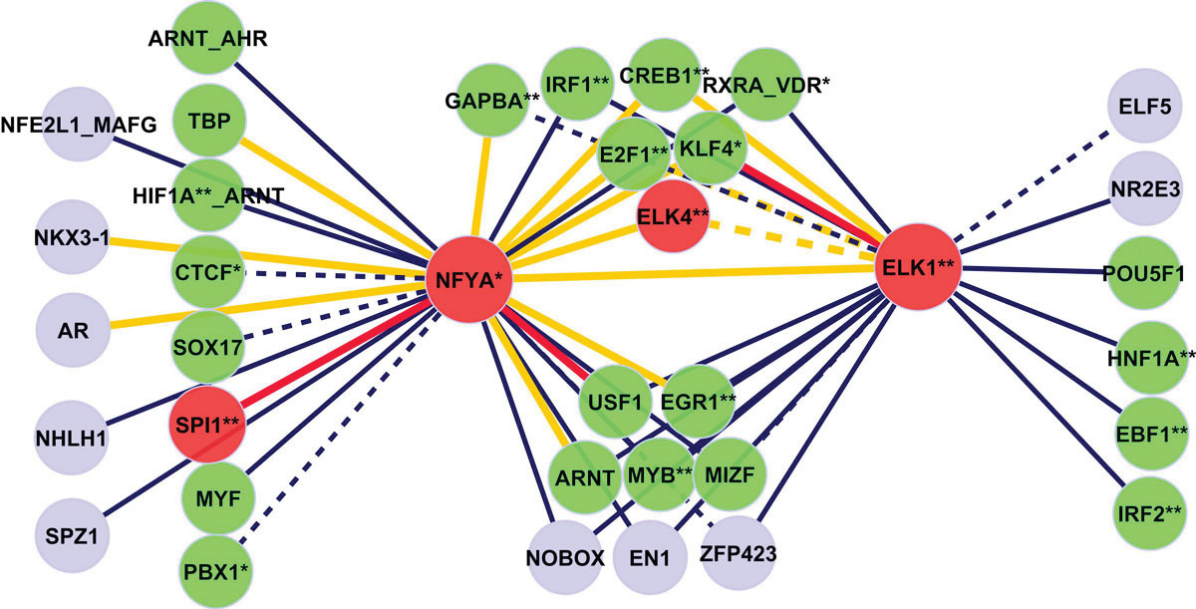
**Predicted network of interactions in hematopoietic stem cells.** Network of predicted TF interactions in hematopoietic stem cells based on testing in 3-way contingency tables. Red nodes denote previously known regulators in hematopoiesis, green nodes indicate TFs expressed in hematopoietic stem cells or in bone marrow. TFs with known function in hematopoiesis are labeled with an asterisk. Red and orange edges denote known PPIs and known trios, respectively.

A predicted interaction network with 50 interactions among 36 TFs in hematopoietic stem cells is shown in Figure 7. This network was generated using the interactions with *p *≤ 10^-11 ^because of the large number of specific genes in the hematopoietic stem cells (678) which induce a higher number of predicted significant interactions. The network consists of two subnetworks with two central hubs: ELK1 and NFYA which were classified as facilitator hubs by FANTOM Consortium [9] too. Both TFs together with ELK4 and SPI1 are known regulators in hematopoiesis. Similar to previously analyzed tissues, a majority (72.2%) of predicted interacting factors is expressed directly in the hematopoietic stem cells or in bone marrow [9,32,34]. We predict 4 already known PPIs (ELK1:KLF4, NFYA:ELK4, NFYA:SPI1, NFYA:CREB1) and 12 trio interactions which share one or more common co-factors (BRCA1, SP1, SRF and TP53).

A functional analysis with IPA Ingenuity Systems showed that 18 (CREB1, CTCF, E2F1, EBF1, EGR1, ELK1, ELK4, GABPA, HIF1A, HNF1A, IRF1, IRF2, KLF4, MYB, NFYA, PBX1, RXRA-VDR, SPI1) of the 36 TFs in our network play a role in the hematopoiesis (*p *= 7.19 × 10^-17^); 13 factors (CREB1, E2F1, EBF1, EGR1, ELK1, ELK4, GABPA, HIF1A, HNF1A, IRF1, IRF2, MYB, SPI1) function in the development of lymphocytes and leukocytes (*p *= 1.77 × 10^-11^).

### Comparison of predicted interactions with other computational methods

The findings of our study predict that the gene expression in tissues is regulated by a large number of tissue-specific interactions which are dominated by central regulators. The central hubs detected with our methodology were confirmed by experimental evidence of the FANTOM Consortium [9]. Here, we want to compare our findings in liver, muscle and hematopoietic stem cells with two other computational methods predicting tissue-specific interactions of TFs.

Yu *et al. *[12] predict interactions between TFs using the relative position and co-occurrence of their binding sites in promoters of tissue-specific genes. For their analysis the PWMs from TRANSFAC database were used. We have identified 11 (HNF1:NFIL3, PBX1:HNF1, HNF4:HNF1, HNF4A:HNF1, HNF1:FOXC1, CEBPA:HNF1, FOXD3:HNF1, HNF1:NKX2-2, HNF1:FOXL1, HNF1:NKX3A, RORA1:HNF1) predicted liver-specific interactions from Yu *et al. *in our liver network too, where HNF1:NFIL3 belongs to the top three liver interactions in their publication. HNF1 is the central regulator in liver described by Yu *et al. *which is in agreement with our liver central hubs HNF1A and HNF1B. 8 of our predicted interactions in muscle (MYF:MEF2, TBP:MEF2, SRF:MEF2, SRF:TBP, RREB1:MEF2, PAX2:MEF2, NF-kappaB:MEF2, TBP:TFAP2A) could be found in the muscle-specific network from Yu *et al*., where MYF:MEF2 is one of the top three interactions. Here, the central regulator is MEF2 which corresponds to our central hub in muscle MEF2A. Since Yu *et al. *do not analyze the interactions in hematopoietic stem cells, direct comparison is not possible. We have therefore examined bone marrow which is the most related tissue including hematopoietic stem cells. 5 predicted interactions (ELK1:GABPA, ELK1:CREB1, ELK1:NFY, ELK1:MYB1, NFY:VDR) from our network could be found in the interacting TF pairs in bone marrow described by Yu *et al*. Hu and Gallo [13] employ a functional conservation approach to predict interacting TFs from tissue-expressed genes. We could identify only two of our predicted TF pairs in liver (HNF1:PAX4, HNF1:SRY) and one TF pair in skeletal muscle (PAX:TBP). One reason for the small overlap may be the different predicted central regulators in tissues. The liver hubs in Hu and Gallo are CEBP, HNF3, and HNF4 whereas our main liver hubs are HNF1A, HNF1B and HNF4A. Our central hub in muscle MEF2A does not occur in the muscle-specific network of Hu and Gallo. The agreement of predictions between Hu and Gallo and Yu *et al. *is very low too.

We see two reasons why the overlap of our interactions and those from Yu *et al. *is much larger than in comparison to Hu and Gallo [13]. First, we use the same set of tissue-specific genes as Yu *et al. *Second, predictions of Yu *et al. *are much more numerous (e.g. 1052 for muscle and 202 for liver) such that the chance to find some common TF pairs is much higher.

## Conclusion

Tissue-specific gene expression is regulated by interactions of multiple transcription factors. To better understand how cells in tissues and developmental states achieve their specificity, the identification of interacting TFs regulating together the expression of their target genes is necessary. Previous computational studies were based either on common sequence features of promoters [10-12] or on function conservation of interacting TFs [13]. Although these studies make plausible predictions, the mechanisms controlling tissue specific gene expression are still not fully understood.

In this study, we presented a new method predicting interactions between TFs. We used the predicted binding affinity information for single TF on promoters and compared the ranked lists of the target genes for all pairs of studied TFs. To identify the interacting pairs in a tissue, tissue specificity information of the target genes was included. We applied statistical testing in 3-way contingency tables to predict TF interactions. The number of interactions between TFs with similar binding sites in our prediction was reduced by focusing on TF pairs with nonsimilar motifs. In total, we have identified 1079 significant TF pairs in 22 human tissues, altogether 767 unique TF pairs. The majority (86.6%) of TF pairs found had nonsimilar motifs. The validity of discovered tissue-specific TF pairs was demonstrated by both known protein-protein interactions and the tissue expression of TFs. We have shown that known protein-protein interactions are enriched (1.8- and 6.8-fold) in the selected candidates with and without tissue specification, respectively. The majority (60 - 70%) of predicted tissue-specific factors were found to be expressed in the studied tissue.

All tissue-specific factors were found just by the selection criterion from the statistical test, without any knowledge about their functions in human tissues. Furthermore, we have identified significantly enriched gene functions related to the examined tissue which support the hypothesis of the regulatory function of these TFs in the tissue. Our predicted networks in human tissues are characterized by one or two central regulators with a high number of interactions. These central hubs correspond to factors such as HNF1A, HNF1B and HNF4A in liver or MEF2A and TBP in muscle or NFYA and ELK1 in hematopoietic stem cells. These have known regulatory function in the studied tissue and an experimentally validated specifier/facilitator hub function by FANTOM Consortium [9]. Despite the successful predictions of novel pairs of interacting TFs, our method could be improved. In general, TFs with very similar motifs (which we excluded from our prediction) can in reality jointly bind to the DNA sequence and regulate the transcription of the target gene. However, our method is not able to distinguish between joint binding of both TFs and binding of a single TF for such similar TFBS. Currently, we use a simple definition of promoter regions. We could theoretically achieve much higher accuracy by using open chromatin regions for various cell types. For our predictions, we have used the groups of genes which are specific for the whole tissue. In general, many mammalian tissues are highly heterogeneous and consist of different types of cells which could be regulated by different combinations of TFs. Including cell-type-specific genes would improve the accuracy of predicted interactions, but since the cell-type groups include smaller numbers of specific genes, the probability of having common specific genes at the top of the ranked lists will be even smaller. A future experimental validation would provide a measure of the specificity and sensitivity of our predictions. Our findings have shown that comparing the ranked lists of target genes results in plausible predictions of interacting TFs in human tissues.

## Competing interests

The authors declare that they have no competing interests.

## Authors' contributions

MV initiated and designed the study, AM performed the data analysis and the validation of predicted interactions. Both authors wrote the manuscript.

## Supplementary Material

Additional file 1 **Table of all predicted interactions by tissue.** Table with all predicted tissue-specific TF pairs with their motif similarity, *p*-value from the 3-way-contingency table test, shared co-factors and PPI database evidence. Click here for file

Additional file 2 **Smooth scatterplot of *p*-values of the 3-way contingency table test in liver and motif similarity measure.** Logarithm of the *p*-values of the 3-way contingency table test for TF pairs in liver (vertical axis) vs. motif similarity measure *S*^max ^(horizontal axis). Red points and orange triangles denote experimentally shown PPIs and trios with a known interacting co-factor, respectively. Click here for file
