# Supplementary figures and images for: Detection of interacting transcription factors in human tissues using predicted DNA binding affinity
Source: BMC Genomics. 2012 Jan 17;13(Suppl 1):S2. doi: 10.1186/1471-2164-13-S1-S2 (PMC3583127; doi:10.1186/1471-2164-13-S1-S2)

# 3-way contingency table test for liver

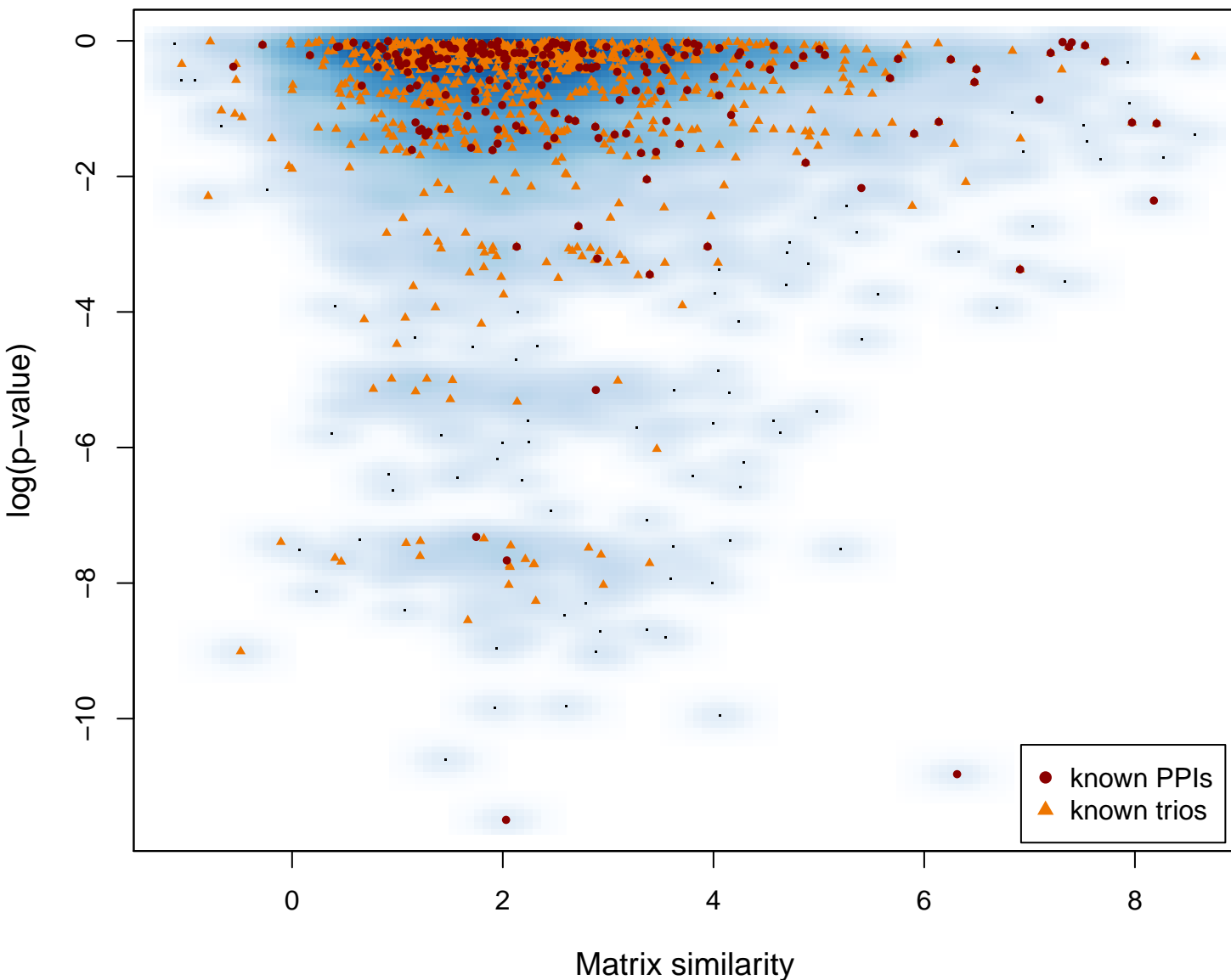

Supplement: Additional file 2 — Smooth scatterplot of p-values of the 3-way contingency table test in liver and motif similarity measure. Logarithm of the p-values of the 3-way contingency table test for TF pairs in liver (vertical axis) vs. motif similarity measure Smax (horizontal axis). Red points and orange triangles denote experimentally shown PPIs and trios with a known interacting co-factor, respectively. [file 1471-2164-13-S1-S2-S2.pdf]
